# From Affective Experience to Motivated Action: Tracking Reward-Seeking and Punishment-Avoidant Behaviour in Real-Life

**DOI:** 10.1371/journal.pone.0129722

**Published:** 2015-06-18

**Authors:** Marieke Wichers, Zuzana Kasanova, Jindra Bakker, Evert Thiery, Catherine Derom, Nele Jacobs, Jim van Os

**Affiliations:** 1 Department of Psychiatry and Psychology, South Limburg Mental Health Research and Teaching Network, EURON, Maastricht University, Maastricht, The Netherlands; 2 Interdisciplinary Center for Psychopathology and Emotion regulation (ICPE), department of Psychiatry, University medical center Groningen (UMCG), Groningen, The Netherlands; 3 Department of Psychiatry and Neuropsychology, University Hospital Ghent, Ghent, Belgium; 4 Department of Neurology, Ghent University Hospital, Ghent, Belgium; 5 Department of Human Genetics, University Hospital Gasthuisberg, Katholieke Universiteit Leuven, B-3000 Leuven, Belgium; 6 Faculty of Psychology, Open University of the Netherlands, P.O box 2960, 6401 DL Heerlen, The Netherlands; 7 Division of Psychological Medicine, Institute of Psychiatry, London SE5 8AF, United Kingdom; UNC Chapel Hill, UNITED STATES

## Abstract

Many of the decisions and actions in everyday life result from implicit learning processes. Important to psychopathology are, for example, implicit reward-seeking and punishment-avoidant learning processes. It is known that when specific actions get associated with a rewarding experience, such as positive emotions, that this will increase the likelihood that an organism will engage in similar actions in the future. Similarly, when actions get associated with punishing experiences, such as negative emotions, this may reduce the likelihood that the organism will engage in similar actions in the future. This study examines whether we can observe these implicit processes prospectively in the flow of daily life. If such processes take place then we expect that current behaviour can be predicted by how similar behaviour was experienced (in terms of positive and negative affect) at previous measurement moments. This was examined in a sample of 621 female individuals that had participated in an Experience Sampling data collection. Measures of affect and behaviour were collected at 10 semi-random moments of the day for 5 consecutive days. It was examined whether affective experience that was paired with certain behaviours (physical activity and social context) at previous measurements modified the likelihood to show similar behaviours at next measurement moments. Analyses were performed both at the level of observations (a time scale with units of ± 90 min) and at day level (a time scale with units of 24 h). As expected, we found that affect indeed moderated the extent to which previous behaviour predicted similar behaviour later in time, at both beep- and day-level. This study showed that it is feasible to track reward-seeking and punishment-avoidant behaviour prospectively in humans in the flow of daily life. This opens up a new toolbox to examine processes determining goal-oriented behaviour in relation to psychopathology in humans.

## Introduction

Many of the decisions and actions in everyday life result from learning processes that are guided by interaction with environmental cues. The capacity to form and continuously update internal representations of stimulus-response associations allows one to select the most advantageous response from a repertoire, and to accurately predict its outcome [1–3]. These implicit associative learning processes are therefore critical mediators of daily life decisions and future course of actions. Consider the example of Peter and Christine who both join the athletics club, in Peter’s case with the goal of becoming a long-distance runner and in Christine’s to make new friends. Peter is tall, slim and fit which predisposes him to excel in endurance activities. He enjoys the training sessions, does not experience a lot of unpleasant physical sensations and improves quickly, soon becoming one of the best runners in the group. As a result, Peter’s repeated exposures to positive outcomes motivate him to keep training. Christine, on the other hand, who is a little shy, sensitive and less interested in running, welcomes the opportunity to join her new teammates in a party. She enjoys the party until one of the girls makes a critical remark about her clothes. She feels very awkward goes home early and skips some practices. When she is invited again to a similar party by her teammates, she declines, until eventually quitting the team. Although seemingly very different, both Peter’s and Christine’s behaviour was governed by two essential mechanisms of operant conditioning [3–6]. Positive reinforcement learning is the process through which new stimuli acquire motivational salience by virtue of being associated with positive emotions, thus becoming rewards. Rewards have, by definition, the potential to increase the likelihood that an organism will engage in actions previously associated with positive affective state [6], as demonstrated by Peter’s case. In this example Peter experiences reward which gets associated with his behaviour of being physically active. This may positively influence Peter’s motivation to repeat this behaviour in the future. Punishment learning, on the other hand, is necessary to avoid aversive outcomes [5,7]. Aversive stimuli decrease the frequency of behaviour linked to negative affective states [6], such as those in Christine’s situation. Christine experiences negative affect which gets paired with being in the above described social context. This may influences her social behaviour, i.e. her choice to avoid those situations in the future. In both examples, the learned associations between (un)pleasant affective experiences and the individual’s behaviour play an important role in motivating future choices to maintain or extinguish the pertinent behaviour. Associative learning processes, therefore, allow for a flexible adaptation of behaviour by guiding organisms in decisions of approach and avoidance of daily life situations, and ultimately prompt and maintain goal-oriented behaviour [6]. The fact that the neural circuits governing associative learning are present even in organisms with the most primitive neural networks [8] demonstrates the importance of this mechanism in survival and evolution.

Experimental studies have shown evidence for positive and negative experiences modifying behaviour in humans [5,9–13]. For example, positive self-talk and self-esteem can be increased by pairing images of a person’s own body with positive stimuli that signal social acceptance [11]. In addition, the unconscious tendency to consume alcohol has been found to decrease following pairing of the alcohol cue with an aversive outcome [9]. Importantly, inadequate reward and punishment learning may play a key role in many forms of deviant behaviour [3,5,14]. For example, whereas impaired punishment learning has been associated with increased risk-taking behaviour and gambling [15], enhanced punishment-avoidance is thought to be related to the acquisition of pain-related fear [12] and in the withdrawal from social situations. Similarly, abnormally high tendency for reward-seeking behaviour is linked with the risk for developing an addiction [9] and eating disorders [16], while suboptimal reward-driven behaviour is believed to be associated with anhedonia, avolition and depression [17,18].

Despite the importance of reward and punishment learning in human behaviour and their putative relevance to mental health, to date, incentive-driven responding has not been studied prospectively in the flow of daily life. This endeavour would provide an ecologically valid assessment of the extent to which affective experience motivates action, with the potential of uncovering real-life behavioural patterns linked to compromised mental health. Physical and social behaviour, as illustrated by the above described two examples, are important types of behaviour that are, in part, controlled by associative learning processes. Furthermore these behaviours have relevance to psychopathology and can be measured using momentary assessment techniques. In the current manuscript we will therefore focus on these two types of behaviour.

Momentary assessment techniques [19–27], enabling the researcher to capture the film rather than a snapshot of daily life reality [28–31], are ideally suited to examine the subtle temporal associations between affect and daily life behaviour over the course of the day(s). Therefore, we can use this method to detect patterns of reward-seeking and punishment-avoidant behaviour by examining whether rewarding or punishing affective states occurring in a certain daily life context, modify the likelihood of engaging in similar daily life contexts in the near future. If we can show that such rewarding or punishing affective experiences influence the likelihood that certain behaviour is repeated more or less often, respectively, then this would support the idea that we can detect the associative learning processes that motivate future behaviour.

The aim of this study is to examine whether it is possible to prospectively track the propagation of reward and punishment-driven behaviour in humans using momentary assessment methodology. For this purpose it was examined, using multilevel analyses, whether affective experience that occurred in the context of specific behaviours at previous measurements (i.e. at t-2 and t-1) modified the likelihood to show similar behaviour at next measurement moments (at time t). In order to obtain initial proof-of-principle, this study focuses on two frequently occurring daily life behaviours which are relevant to mental health states: social context and physical activity. To this end, data pertaining to a general population sample of 621 individuals who participated in an Experience Sampling Method (ESM) study were analysed.

## Methods

### Sample

The study sample consisted of 621 female subjects from the general population, who were part of twin pairs or were sisters of twin pairs living in Flanders (Belgium). Their age was between 18 and 46 years. Of the 621 subjects, 610 participated in the ESM study. Most participants were recruited from the East-Flanders Prospective Twin Survey, a population-based survey prospectively recording all multiple births in the province of East-Flanders since 1964 [32,33]. Selection criteria were being female and being over 18 years of age. Participants received a financial compensation for participation of 500 Belgium Francs (which is the equivalent of approximately 12,5 euros). Although subjects were twins, the current hypothesis did not require twin methodology. The project was approved by the Medical Ethics Committee of the K.U. Leuven under the number B3222010766. All participants gave written informed consent. Originally, the study was designed to assess stress-sensitivity in daily life; taking into consideration evidence for qualitative differences in the type of environmental stressors that are associated with depression in men and women [34,35], the study was designed to include females only. Mean age of the participants was 27 years (SD: 7.6 years, range (18–46). Sixty-five percent had a college or university degree, 33% completed secondary education and 2% had primary education only. The majority was currently employed (61.3% employed, 33.8% student, 2.2% unemployed, 2.3% homemaker and 0.4% sick leave).

### Experience Sampling Method

The experience sampling method (ESM) [36] was used to conduct momentary assessments on 5 consecutive days. Subjects received a digital wristwatch and a set of ESM self-assessment forms collated in a booklet for each day. The wristwatch was programmed to emit a signal (“beep”) at an unpredictable moment in each of ten 90-minute time blocks between 7:30 and 22:30. The study uses a semi-random beep design in order to prevent anticipatory behaviour of participants, but also used the constraint that no two signals could occur within 15 minutes of each other [37]. Beep intervals did not overlap, thus ensuring that the first beep of the day was always earlier in time than the second and the second earlier than the third etc. The semi-random beep design was set up in a way that every moment of the day had an equal likelihood of being sampled.

Participants were aware that 10 beep signals would occur in a day between 7.30 and 22.30. They were instructed to fill out the ESM diary directly after each beep signal. The diary consisted of short questions on the current affect, behaviour and appraisals of the current situation (see below). All self-assessments were rated on 7-point Likert scales. Trained research assistants with ample experience in momentary assessment technology explained the ESM procedure to the participants during an initial briefing session, and a practice form was completed to confirm that subjects were able to understand the 7-point Likert scale. Subjects could call a telephone number in case they had questions or problems during the ESM sampling period. Subjects were instructed to complete their reports immediately after the beep, thus minimizing memory distortion, and to record the time at which they completed the form. The time at which subjects completed the report was compared to the actual time of the beep that signaled that it was time to fill out the report. In this way it was checked whether reports were filled out within 15 minutes. All reports not filled out within 15 min after the beep were excluded from the analysis, since previous work [22] has shown that reports completed after this interval are less reliable and consequently less valid. In addition, subjects with less than 17 valid reports (out of 50) were excluded from the analysis, as previous work has shown that measures of individuals with less than 30% of completed reports are less reliable [22]. Compliance to the ESM protocol was very high (96.4%) [38].

### Measurements

#### Momentary affect

Momentary affective states were assessed with 15 adjectives rated on 7-point likert scales. The choice of the ESM affect items was guided in part by the PANAS questionnaire [39] and in part by the results of previous ESM studies (selecting items with high loadings on NA and PA latent factors and sufficient within-person variability). A Factor analysis on the affect items identified two affect factors with eigenvalue >1. Ratings on the items ‘insecure’, ‘lonely’, ‘anxious’, ‘low’, ‘guilty’ and ‘suspicious’—weighted for factor loadings—were averaged to form NA (respective loadings were: 0.71, 0.60, 0.66, 0.68, 0.61, 0.64). The weighted average of ratings on ‘cheerful’, ‘content’, ‘energetic’, ‘enthusiastic’ formed PA (respective loadings were: 0.84, 0.71, 0.84, 0.83). The strongest cross-loading was -0.13 (loading of ‘content’ on NA factor). Five affect items did not load specifically on one of these factors. These were ‘tired’, ‘relaxed’, ‘irritated’, ‘sad’ and ‘calm’. Conform previous publications [40,41] that used this sample these five items were not used in the variables that measure NA and PA.

#### Appraisal of social context

Appraisal of the social context was assessed by asking participants whether or not they were alone at the time of the beep. If not alone, they were asked whether they liked the company they were in at that moment. This was rated on a 7-point likert scale (from ‘not at all’ (1) to ‘very much’ (7)).

#### Physical activity

ESM physical activity was assessed with a single item asking subjects how physically active they had been since the last beep, rated on a 7-point likert scale. During the ESM briefing, participants were instructed on how to score their level of physical activity. To this end, they were given an indication of the level of activity that corresponded to the score on the 7-point likert scale. Examples were provided as anchor points as follows: a score of ‘1’ corresponds to the level of physical activity of ‘resting’; ‘2’ corresponds to ‘sitting’; ‘3’ to ‘walking’; ‘4’ to ‘household chores such as vacuum cleaning’; ‘5’ to ‘biking’; ‘6’ to ‘playing tennis’ and ‘7’ to ‘running’. These anchor points were also provided in the ESM self-assessment forms that subjects completed at each beep. For instance, for each physical activity approximately equalling the level of physical activity associated with vacuum cleaning, participants rated a score of 4 on the 7-point likert scale.

### Statistical analysis

ESM data have a hierarchical structure in which ESM observations (level 1) are clustered within participants (level 2). STATA 11.0 [42] was used to conduct multilevel regression analyses. Time-lagged multilevel analyses were used to examine reward- and punishment-based behaviour prospectively over time.

**Fig 1:**
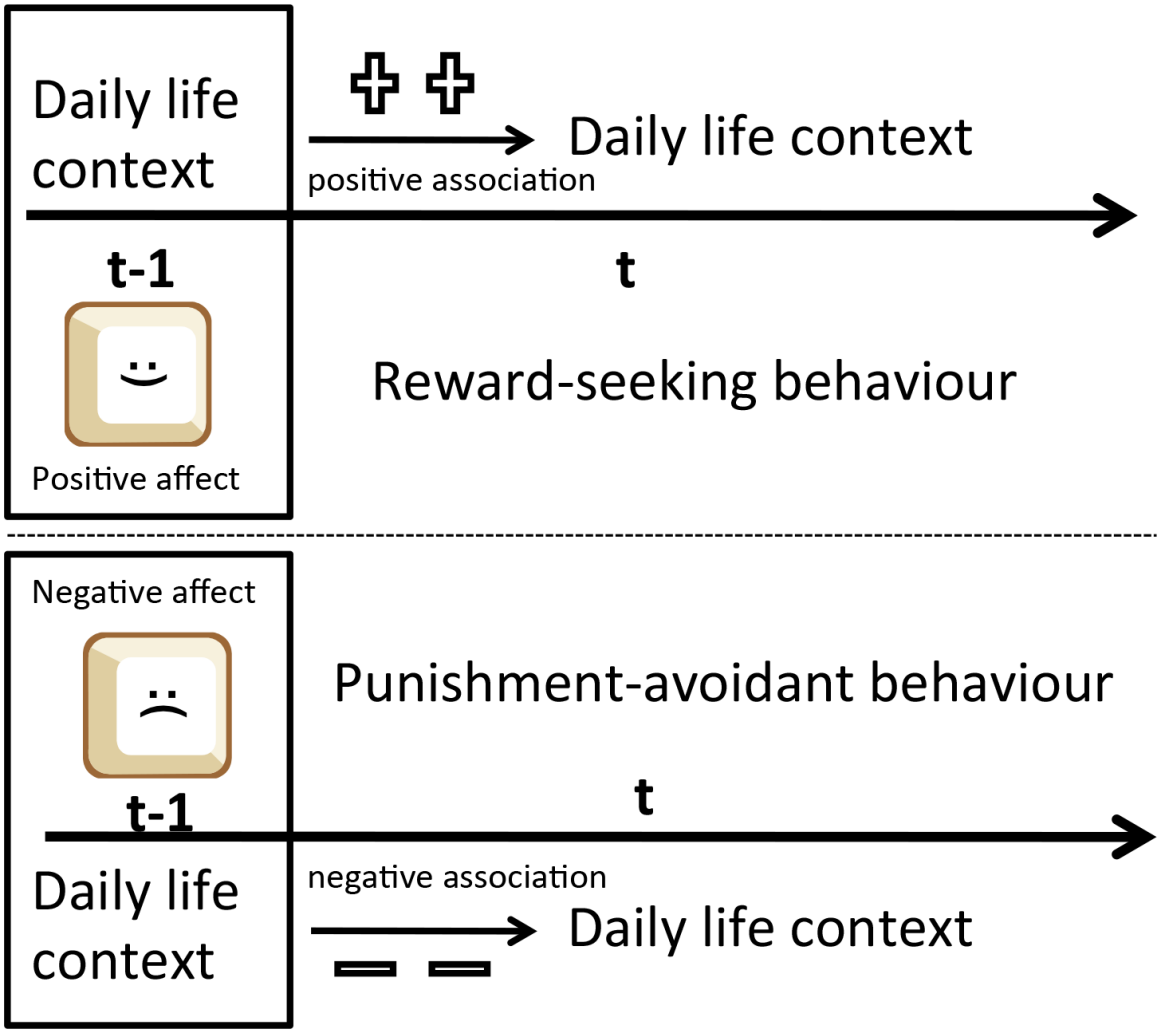
schematically depicts how reward- and punishment-related behaviour is operationally defined in this article. Reward-related behaviour is conceptualized as the daily life behaviour (such as being in pleasant company or engaging in physical activity) that increases in frequency at time t upon being associated with positive affect at time t-1. Punishment-related behaviour is conceptualized as the daily life behaviour the frequency of which decreases at time t upon being associated with negative affect at time t-1. Time lags t-1 and t-2 are examined in this study where t represents the time lag of one beep moment (± 90 minutes) or the time lag of one day.

Reward-related behaviour is the result of an associative process in which positive affective experience is paired with a behaviour, and the frequency of this behaviour increases. Assuming that positive affect is rewarding, reward-driven behaviour was operationalized in ESM as the moderating effect of PA at (t-1) on the probability that behaviour at (t-1) was repeated at (t) (Fig 1). Punishment-related behaviour represents the opposite process in which the occurrence of the behaviour previously associated with negative affective experience decreases. Assuming that negative affect is punishing, punishment-related behaviour was thus operationalized in ESM as the moderating effect of NA at (t-1) on the probability that behaviour at (t-1) was repeated at (t) (Fig 1). Mathematically, the i-th momentary observation score of subject j is modelled as follows:

Behaviour_ij t_ = β_0_ + β_1_ affect_ij t-1_ + β_2_ behaviour_ij t-1_ + β_3_ affect_ij t-1_*behaviour_ij t-1_ ζ._j_ ε_ij_. Here, ζ_j_ represents the subject’s deviation from the overall mean (random intercept).

In order to obtain a regression model that purely tests within-subject effects, the between-subject regression was subtracted from the original regression model, as follows: Behaviour_ij t_ behaviour._j t_ = β_1_ (affect_ij t-1_ - affect._j t-1_) + β_2_ (behaviour_ij t-1_ − behaviour._j t-1_) + β_3_ (affect_ij t-1_*behaviour_ij t-1_- affect._j t-1_*behaviour._j t-1_) +ε_ij_ - ε._j_. In other words, from each predictor in the regression model we subtracted the subject mean, leaving only pure within-subject effects. These regression analyses were performed using the XTREG command combined with the fixed effects (*fe)* option. Since the *fe* option is available only for multilevel analyses with no more than one extra level, clustering within twin pairs was accounted for by adding twin pair id as a covariate. The above example equations describe the model including one lag effect. The regressions performed in this study included 2 lags.

Generalisability over different time frames: In addition to beep-level (t duration = ± 90 min), reward-related and punishment-related behaviours were also explored at day-level (t duration = 1 day). For the day-level analyses, beep moment results of affect, behaviour and the interaction thereof were averaged per beep over the day. These analyses thus examined whether the average of the repeated (maximum 10) combinations of affect and behaviour at day (t-1) impacted on the average behaviour on beep moments at day (t).

Specificity of effects: Additionally, cross-context associations were added to examine the specificity of the effect. If time-lagged effects across the different behavioural contexts are equally strong as those within behavioural contexts this would be indicative for non-specific effects of affect impacting on future behaviour in general. If, on the other hand, within-context effects are much stronger than cross-context effects, then this supports the idea that we detected behaviour-specific processes of associative learning.

## Results

Of the 610 subjects who participated in the ESM study, 31 were excluded because they had too few (less than 30%) beeps with valid responses. Of the 579 remaining subjects, 12 participants had been part of the first pilot data collection. Some of the PA items that were present in the final diary were not present in the pilot diary. As some of the PA items were missing in the pilot sample, the total PA scores could not be computed for these 12 participants. Another 4 participants had no intra-individual variations in or no consecutive measurement moments for physical activity, and were therefore excluded from the analysis. In the various analyses, the total number of beep-level measurements varied between 9,990 and 12,228. The total number of day-level measurements varied between 1,642 and 1,693. The data file and a short description of the variables are available in the supporting information files (see S1 Dataset and S1 Text). In Table 1 the correlations at observation-level are shown between NA, PA, (un)pleasantness of company and physical activity.

**Table 1:** shows the correlations, at observation-level, between negative affect, positive affect, (un)pleasantness of company and physical activity.

	Negative affect	Positive affect	(Un)pleasantness of company	Physical activity
**Negative affect**	-			
**Positive affect**	-0,35	-		
**(Un)pleasant ness of company**	-0,21	0,30	-	
**Physical activity**	0,007	0,11	-0,02	-

### Reward-related behaviour

Table 2 shows the regression analyses results regarding temporal patterns of reward-related behaviour for beep and day-level. The beep-level analyses refer to multilevel time-lagged analyses in which time units are used from beep moment to beep moment (which are on average 90 minutes apart from each other). Day-level analyses refer to similar multilevel time-lagged analyses, but now using time units of 24 hours.

**Table 2:** Reward-related behaviour: Regression coefficients and p-values of the *interaction effects* (PA x behaviour at lags 1 and 2 on behaviour at (t)) at beep and day level.

BEEP-LEVEL	Lag 1		Lag 2	
*Within-context effects*	*b-coefficient*	*p-value*	*b-coefficient*	*p-value*
PA_t-n_ ^φ^ x pleasant company_t-n_ on pleasant company_t_	0.016	0.037*	0.008	0.287
PA_t-n_ x physical activity_t-n_ on physical activity_t_	0.022	0.012*	0.002	0.808
*Cross-context effects*				
PA_t-n_ x pleasant company_t-n_ on physical activity_t_	0.003	0.678	0.001	0.906
PA_t-n_ x physical activity_t-n_ on pleasant company_t_	0.009	0.422	-0.012	0.279
DAY-LEVEL				
*Within-context effects*	*b-coefficient*	*p-value*	*b-coefficient*	*p-value*
(PA x pleasant company)_t-n_ on pleasant company_t_	0.091	<0.001*	0.029	0.247
(PA x physical activity)_t-n_ on physical activity_t_	-0.014	0.528	0.023	0.288
*Cross-context effects*				
(PA x pleasant company)_t-n_ on physical activity_t_	-0.048	0.025*	-0.062	0.004*
(PA x physical activity)_t-n_ on pleasant company_t_	-0.057	0.053	-0.086	0.003*

^φ^n is either 1 (in case of 1 lag) or 2 (in case of 2 lags)

* significant finding (α < 0.05).

#### Beep-level

**Fig 2:**
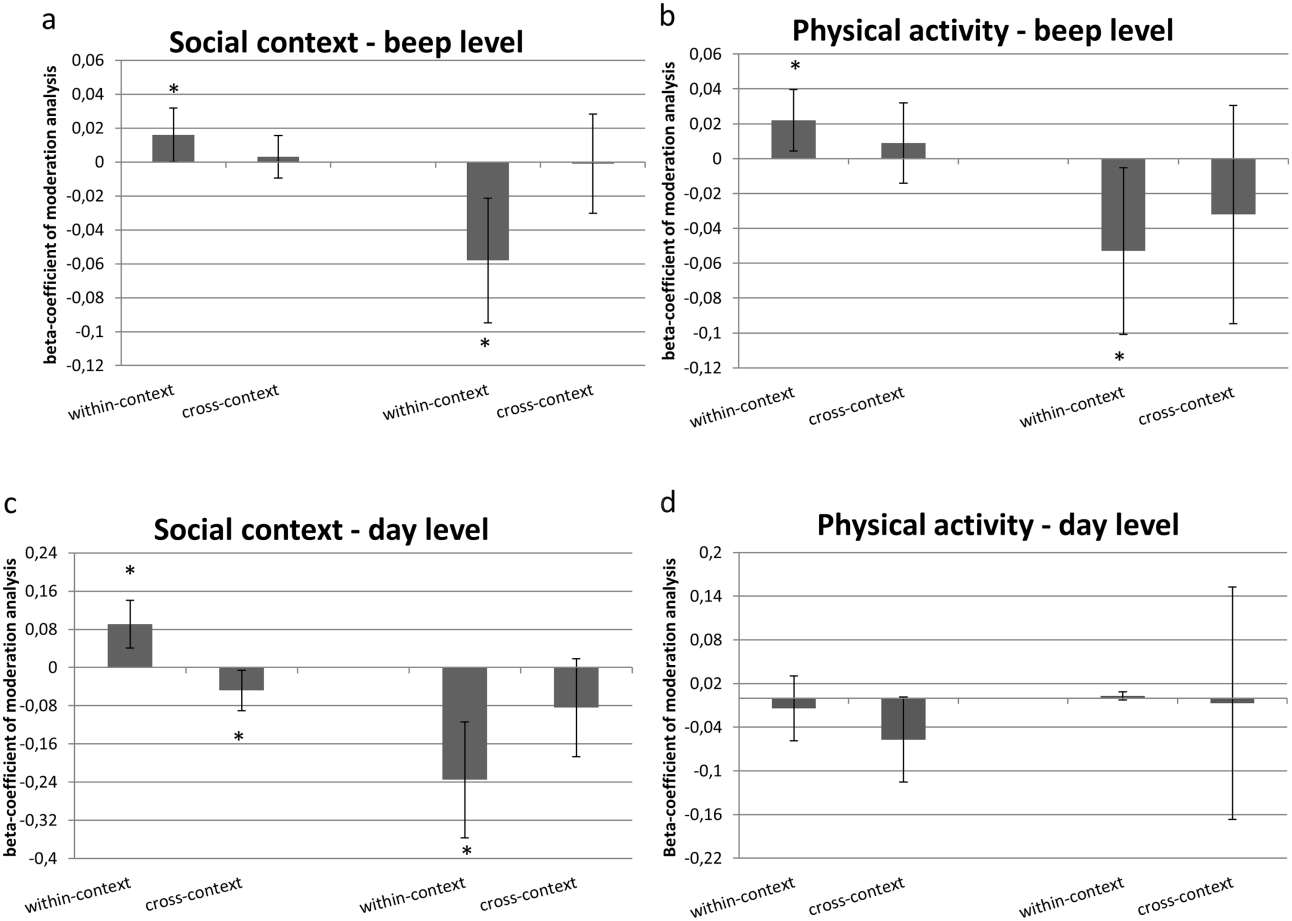
shows beta-coefficients for reward- and punishment-related behaviour at beep level and day level for lag t-1. The first two bars in each Fig. represent the analyses with positive affect at t-1 as the moderator of the time-lagged association between daily contexts and the second two bars represent the analyses with negative affect at t-1 as the moderator of these analyses. Each first bar is the within-context association (the same context is predictor at t-1 and outcome measure at t. This bar thus reflects the extent to which affect valence moderates the association between the analysed behaviour at time t-1 on the same behaviour at time t. The second bar refers to the cross-context association: the extent to which affect moderates the time-lagged association *across* daily contexts (for example: social context as the predictor and physical activity as the outcome). Error bars represent confidence intervals. The Fig.s show that, as hypothesized, positive and negative affect both significantly moderate the impact of behaviour at time t-1 on similar behaviour at time t, except for the analyses regarding physical activity at day-level (Fig 2d).

Regarding the beep-level analyses, significant within-context interaction effects were found for both behaviours: engaging in physical activity and being in pleasant company. More specifically, PA moderated the effect of physical activity at beep (t-1) on physical activity at beep (t). The same was true for being in pleasant company. Significant effects were observed only for associations over one lag. No significant cross-context effects (e.g. from physical activity to pleasant company or vice versa) were found (see Table 2 and Fig 2). Thus, positive affect indeed moderated the likelihood of recurrence of active and social behaviour in the expected direction. Furthermore, the fact that no cross-context effects were observed makes it unlikely that observed effects represent non-specific effects of affect impacting on future behaviour in general.

#### Day-level

**Fig 3:**
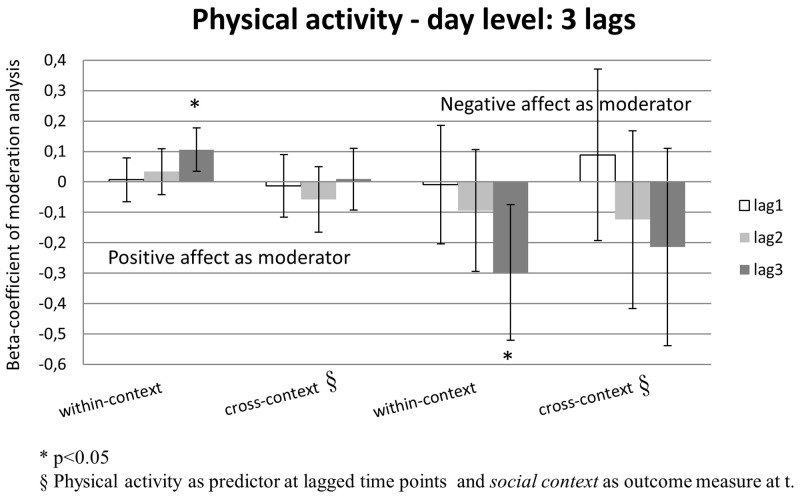
shows the beta-coefficients of reward- and punishment-based responding for physical activity for three lag moments (t-1, t-2 and t-3). A stronger association of physical activity with positive affect at day t-3, corrected for effects at day t-2 and day t-1, increases physical activity at day t. Similarly, a stronger association between physical activity with negative affect at day t-2, corrected for effects at day t-2 and day t-1, decreases physical activity at day t. No significant cross-context effects were found. Error bars represent confidence intervals. This Fig. shows that the hypothesized effects do show up in post-hoc analyses where we corrected not just for 2 but for three time lags (3 days).

PA at day (t-1) moderated the effect of being in pleasant company at day t-1 on being in pleasant company at day (t) (one lag). This effect was not found for day (t-2) on day (t)(two lags). Thus, higher levels of PA in the context of pleasant company increased the level of pleasant company on the next day. For physical activity, no significant interaction effects were found for lag 1 and 2 (see Table 2; Fig 2). However, strongly negative *main effects* were observed regarding physical activity at day (t-1) and day (t-2) on day (t) (B = -0.28, p<0.001 and B = -0.24, p<0.001, respectively). This suggests a sinusoid pattern in which high levels of physical activity on one day are associated with less activity the next two days. Therefore, more lags might be necessary to reveal a potentially delayed effect of PA during physical activity and the occurrence of physical activity in the future. In order to examine such pattern, a *post-hoc* test was carried out of the effects of an additional lag. Corrected for effects at lag 1 and 2, lag 3 revealed a significant interaction effect where PA positively moderated the effect of physical activity at day (t-3) on physical activity at day (t) (B = 0.106, p = 0.003; Fig 3). No positive moderation effects of PA were found in cross-context analyses. In contrast, negative moderation was observed (see Table 2). Thus, also at day-level positive affect moderated the likelihood of recurrence of active and social behaviour in the expected direction.

### Punishment-related behaviour

#### Beep-level

NA at beep (t-1) negatively moderated the effect of unpleasant company at beep (t-1) on unpleasant company at beep (t). Thus, higher levels of NA decreased the effect of being in unpleasant company at beep (t-1) on being in unpleasant company at beep (t). No interaction effect of beep (t-2) was observed. A similar effect was found for physical activity in that NA at beep (t-1) negatively moderated the effect of physical activity at beep (t-1) on physical activity at beep (t). No significant negative moderation was observed for cross-context interaction effects (see Table 3 for effect sizes and p-values of the interaction effects). Thus, as expected, negative affect negatively moderated the likelihood of recurrence of active and social behaviour. Again, as expected, these effects were only present for within-context and not for cross-context analyses.

**Table 3:** Punishment-related behaviour: Regression coefficients and p-values of the *interaction effects* (NA x behaviour at lags 1 and 2 on behaviour at (t)) at beep and day level.

BEEP-LEVEL	Lag 1		Lag 2	
*Within-context effects*	*b-coefficient*	*p-value*	*b-coefficient*	*p-value*
NA_t-n_ ^φ^ x unpleasant company_t-n_ on unpleasant company_t_	-0.058	0.002*	0.004	0.812
NA_t-n_ x physical activity_t-n_ on physical activity_t_	-0.053	0.027*	-0.019	0.409
*Cross-context effects*				
NA_t-n_ x unpleasant company_t-n_ on physical activity_t_	-0.001	0.969	-0.023	0.115
NA_t-n_ x physical activity_t-n_ on unpleasant company_t_	-0.032	0.314	-0.004	0.900
DAY-LEVEL				
*Within-context effects*	*b-coefficient*	*p-value*	*b-coefficient*	*p-value*
(NA x unpleasant company)_t-n_ on unpleasant company_t_	-0.235	<0.001	0.005	0.941
(NA x physical activity)_t-n_ on physical activity_t_	0.003	0.960	-0.012	0.849
*Cross-context effects*				
(NA x unpleasant company)_t-n_ on physical activity_t_	-0.084	0.101	-0.001	0.983
(NA x physical activity)_t-n_ on unpleasant company_t_	-0.007	0.931	-0.115	0.172

^φ^n is either 1 (in case of 1 lag) or 2 (in case of 2 lags)

* significant finding (α < 0.05).

#### Day-level

NA at day (t-1) negatively moderated the effect of being in unpleasant company at day (t-1) on being in unpleasant company at day (t). This interaction effect was not found for day (t-2). No significant negative moderation was observed for physical activity (see Table 3). However, similar to the analyses of reward-related behaviour, there were significant negative main effects of physical activity at day (t-1) on physical activity at day (t), possibly obscuring the associative patterns. Therefore, the effect of an extra day-lag was examined *post-hoc*. Results showed that NA negatively moderated the effect of physical activity at day (t-3) on physical activity at day (t) (B = -0.298, p = 0.008 (see Fig 3). No significant cross-context interaction effects were found (see Table 3). Thus, also at day-level negative affect moderated the likelihood of recurrence of active and social behaviour in the expected direction.

## Discussion

The hypothesis that reward and punishment-related behaviour can be prospectively tracked using momentary assessment techniques was confirmed. Both at beep and day level, affect, experienced in relation to certain behavioural contexts, influenced the frequency with which similar behaviour was expressed in the future.

### Social context

As expected, the experience of positive affect in pleasant social contexts increased the extent to which these contexts predicted the occurrence of similar social contexts in the near future at beep and day level (within-context), but did not increase the extent to which pleasant social contexts predicted the occurrence of other activities, such as physical activity (cross-context). At beep level, where measurements are on average 90 minutes apart, this finding could indicate two things. First, it could reflect a tendency to search for new enjoyable company directly following a previous rewarding social experience. Second, the finding may indicate that people tend to prolong the amount of time in a specific social context if it is associated with experience of positive affect. At day level, the latter interpretation is not very likely. Here, the repeated associations between positive affect and pleasant social contexts, derived from all measurement moments during the day together, predicted the frequency of being in similar pleasant company the next day. Day level findings, therefore, might reflect associative processes that involve a more complex integration of multiple response-outcome contingencies to effectively evaluate what future actions may be beneficial in the long-term. It is thought that higher-order cortical processing in the prefrontal and anterior cingulated cortex is involved in decision-making based on the synthesis and evaluation of a large number of previously internalized associations [43–46]. The beep-level effects in this study could be more indicative of short-term responses to immediate affective experience instead of being based on the integration of a long-term history of experiences.

Also, as predicted, negative affect associated with unpleasant social contexts decreased the likelihood of the occurrence of similar social contexts in the near future at both beep- and day-level. Similar to the reasoning above, the beep-level findings could mean either that people avoided new negative social situations after negative social experiences one or two hours earlier that day, or that people tended to shorten their contact with company in which they experienced negative affect. In conclusion, the valence of affective experience associated with social behaviour was found to modify the frequency of occurrence of this behaviour. This effect, however, was detected for one lag, but not for two lags. This means that the pertinent affective state was found to modify behaviour for up to 90 minutes at beep-level and for up to 1 day at day-level.

### Physical activity

As hypothesized, significant within-context and non-significant cross-context effects were observed for physical activity at beep-level. That is, positive affective experience in the context of physical activity predicted increased frequency of occurrence of physical activity at the next measurement moment. Similarly, negative affective experience in the context of physical activity was associated with decreased propensity to engage in physical activity at the next measurement moment. Thus, both reward and punishment contingencies were found to modify behaviour at beep-level. Similar to social context, these effects were only found to hold at one time lag but not at two time lags. In contrast to the social context findings, however, no significant association between affective experience related to physical activity and frequency of future engagement in physical activity was detected at day level. The lack of significant findings at day level is likely caused by the strongly negative association between physical activity on similar activity the next day. Arguably, this pattern may be a demonstration of the natural day-to-day fluctuations in physical activity levels; people tend to be less active during the days following a physically more challenging day, reflecting a compensatory mechanism. It can be hypothesised that these day-to-day fluctuations in activity obscured the effects of the propagation of associative processes, since adding an additional third lag (Fig 3) revealed the expected within- and cross-context effects. It is thus important to find the appropriate level at which associative processes can be modelled for each type of behaviour studied. Behavioural modification regarding physical activity apparently requires examining patterns over multiple days to accurately track the intermittent nature of physical activity and rest.

### Experimental paradigms

Previously, studies have shown evidence of reward and punishment-driven behaviour using experimental paradigms involving monetary rewards or losses. One experiment showed, using a monetary incentive delay task, that the ability to learn to avoid losses was associated with differences in insular sensitivity to anticipated losses and with self-reported levels of anxiety [47]. Another study showed that higher arousal responses at moments of a threat cue-signalling the delivery of a shock- were associated with increased learning of avoidance responses [10]. Also, reward sensitivity assessed experimentally [18,44,48–54] using monetary incentives was found to be modulated by stress, genetic predisposition and anhedonic states. To date, however, it is unknown how experimental findings based on secondary rewards and punishments extrapolate to real-life stimulus-response relationships, and how these manifest themselves in real-life situations, such as social contexts or personal activities. While experimental studies provide valuable insights into psychological and biological mechanisms of relevant traits, momentary assessment techniques may be able to expand on their findings and provide real-life validity to experimental associative processing models. Real life validation of experimental findings would connect existing knowledge on mechanisms of reward- and punishment-driven responding with concrete patterns of daily life sub-optimal goal-oriented behaviour that can be targeted for therapeutic modification. Therefore, future studies should combine experimental and momentary assessment techniques to examine how experimental findings translate to real life behavioural and affective patterns. To our knowledge, only one recent study [55] has combined experimental imaging and momentary assessment techniques. The authors showed that prefrontal stress-related dopamine activity is associated with real life reactivity to stress. The current momentary assessment study is the first to show that the propagation of incentive-based behaviour can be examined in the flow of daily life, paving the way for the first study combining experimental and momentary *associative processing* paradigms.

### Clinical relevance

The findings of the current study showed that behaviour can be modified as a function of the affective experience associated with it, a pattern that was detectable at the micro-level with time lags of approximately 90 minutes, but also at day-level, with time lags of one day. It still needs to be established, though, whether these real-life associative patterns are indeed relevant to psychopathology, and whether their relevance can be modulated by time frame of analysis (beep or day) level. In other words, with respect to psychopathology, it remains to be determined whether it has potential to examine person-specific patterns of behavioural modification on a moment-to-moment or on a day-to-day basis.

However, should these prospectively measured behavioural patterns be involved in the development of psychopathology, then analysing momentary assessment data may represent a new tool to provide person-tailored clinically useful information [30]. Person-specific analyses may reveal person-tailored results regarding reward-seeking and punishment-avoidant behaviour in real life. Such person-specific insights into daily life learning mechanisms may be of direct clinical use as input for therapist-patient contacts [30,56–58].

### Methodological issues

Effect sizes as reported in the current study are small. However, it is generally observed that in ESM studies effect sizes are smaller than effects found using traditional research designs since in ESM effects occur over very small periods of time. These effects represent averaged effects that happen each 90 minutes or each day, depending on the time frame. Cumulatively, these repeated effects seem influential enough to impact on mental health as has been shown in previous studies [59–61].

Second, it is unknown whether the appraisal of company reflects an individual’s active choice to be in the company of certain people, or the subjective interpretation of the social context. If the latter is true, then these findings may reflect an alteration in the *appraisal* of the company, rather than a change in behaviour. This would mean that the previous day’s PA experience during pleasant company would improve the appraisal of company during the next day. This alternative interpretation can mean two things: (i) it reflects non-specific effects of PA. However, since no cross-context effects of PA were found, this is not probable; (ii) it reflects reward-modulated interpretation of daily life context. Either form of reward-driven modification (at the behavioural level or at the level of interpretation of daily life contexts) is clinically relevant. The same line of reasoning can be applied to the analyses on aversive learning.

The data of the current study were collected from 1999 to 2002. No electronic ESM devices were available at that time. However, compliance to the research protocol was electronically monitored in a subgroup of the sample and was determined to be high (see for more details [38]).

The current study used ESM for 5 consecutive days. However, longer periods of sampling (2–3 weeks) are preferable, especially for the modelling at day-level. The development of electronic ESM devices which are less demanding for participants than the paper-and-pencil method, may facilitate extended sampling periods.

Another issue is the use of self-report only. ESM is one of the most accurate ways to measure affect. The experience of affect cannot be reliably estimated using objective measures. However, for physical activity, for example, it would be possible to use objective measures such as actigraphy. Since both self-report and objective measures contain a certain level of noise, the measure of physical activity could be improved by using a combination of both measures.

Finally, the sample included females only. Therefore, results do not necessarily extrapolate to male individuals.

## Supporting Information

S1 Dataset This file includes the variables that are used in the current manuscript. (XLS)

S1 Text This file includes a short description of the variables included in the S1 dataset. (DOCX)
