# Supplementary material for: From Affective Experience to Motivated Action: Tracking Reward-Seeking and Punishment-Avoidant Behaviour in Real-Life
Source: PLoS One. 2015 Jun 18;10(6):e0129722. doi: 10.1371/journal.pone.0129722 (PMC4472779; doi:10.1371/journal.pone.0129722)
Supplement: S1 Text — (DOCX) [file pone.0129722.s002.docx]

**Supporting information of the variables in the data file**

Subjnr: identification number of the participant

Dayno: number of day in ESM study. (day 1 means first day of ESM)

Beepno: number of beep within a day. There were a maximum of 10 beeps to fill out spread over each day.

Idtw: identification number of the twin pair participating in the study

Rang: order of the twins. In each twin pair there is a twin 1 and a twin 2.

Beepcode: unique identification number of observations in the data set

Siaangnm: appraisal of (un)pleasantness of the company ranging from (I do not like this company at all (1) to I like this company very much (7).

Posture: level of physical activity since the last beep (from 1 (very low activity) to 7 (very high activity))

Naw: negative affect score weighted for factor loadings of each negative affect item

Paw : positive affect score weighted for factor loadings of each positive affect item
